# Virtual Reality Clinical Research: Promises and Challenges

**DOI:** 10.2196/10839

**Published:** 2018-10-17

**Authors:** Bernie Garrett, Tarnia Taverner, Diane Gromala, Gordon Tao, Elliott Cordingley, Crystal Sun

**Affiliations:** 1 School of Nursing University of British Columbia Vancouver, BC Canada; 2 School of Interactive Arts and Technology Simon Fraser University Surrey, BC Canada; 3 Rehabilitation Sciences University of British Columbia Vancouver, BC Canada; 4 Faculty of Science University of British Columbia Vancouver, BC Canada

**Keywords:** virtual reality, clinical research, VR standards, VR theory, VR immersion, VR presence

## Abstract

**Background:**

Virtual reality (VR) therapy has been explored as a novel therapeutic approach for numerous health applications, in which three-dimensional virtual environments can be explored in real time. Studies have found positive outcomes for patients using VR for clinical conditions such as anxiety disorders, addictions, phobias, posttraumatic stress disorder, eating disorders, stroke rehabilitation, and for pain management.

**Objective:**

This work aims to highlight key issues in the implementation of clinical research for VR technologies.

**Methods:**

A discussion paper was developed from a narrative review of recent clinical research in the field, and the researchers’ own experiences in conducting VR clinical research with chronic pain patients.

**Results:**

Some of the key issues in implementing clinical VR research include theoretical immaturity, a lack of technical standards, the problems of separating effects of media versus medium, practical in vivo issues, and costs.

**Conclusions:**

Over the last decade, some significant successes have been claimed for the use of VR. Nevertheless, the implementation of clinical VR research outside of the laboratory presents substantial clinical challenges. It is argued that careful attention to addressing these issues in research design and pilot studies are needed in order to make clinical VR research more rigorous and improve the clinical significance of findings.

## Introduction

**Figure 1:**
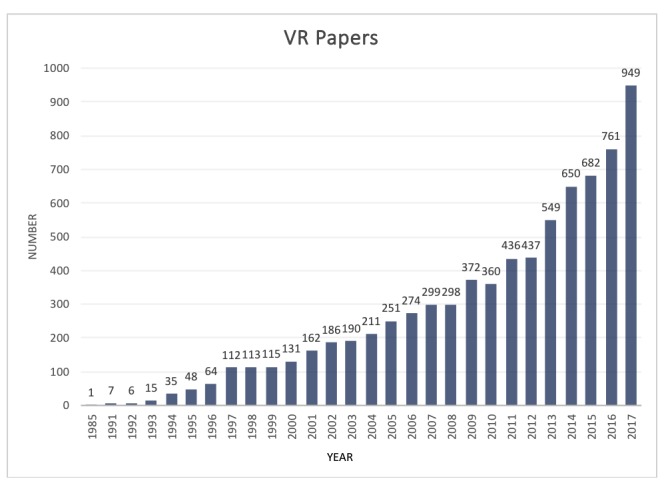
VR clinical application papers published by year (PubMed).

Contemporary research on computer-based virtual reality (VR) dates back to the early 1980s, although devices for presenting stereoscopic imagery (ie, using a slightly different image for each eye) such as the stereoscope started in the 1830s [1]. The exploration of VR use in clinical applications is accelerating rapidly with the advent of more powerful computer and graphics processors capable of rendering real-time three-dimensional (3D) imagery, and the availability of relatively low-cost VR headsets such as the Oculus Rift or HTC Vive (see Figure 1).

As researchers with significant experience in researching VR for clinical applications, we have identified some major issues in the development of clinical VR research. Substantial challenges remain with theoretical ambiguity and immaturity, a lack of technical standards, problems of media versus medium, practical in vivo issues, and economic feasibility.

### Background

There has been rapid growth in the reported use of VR in the treatment of a variety of clinical conditions, such as acute and chronic pain management [2-9], anxiety disorders [10-12], phobias [13-15], posttraumatic stress disorder (PTSD) [16-18], eating disorders [19], autism [20], and rehabilitation [21-26]. Additionally, its use in professional health care education has also been expanding rapidly [22,27-32].

One early clinical application of VR was for the treatment of acrophobia [33]. Graduated exposure to virtual environments with foot bridges, balconies, and a glass elevator were used with a railing placed around the user in the real world for them to hold on to. The intervention was reported as effective. Over the last 20 years, VR clinical applications have expanded to address other phobias and anxiety disorders. The most common approaches in this field have been to model virtual environments after existing exposure therapies using graduated exposure to a VR version of the object or situation that causes distress and use of VR cognitive behavioral therapies [34-37]. For PTSD, virtual environments have been used to simulate complex traumatic scenarios under control to treat war survivors [18,38]. Similarly, VR has been used in the treatment of body image and eating disorders [39-41]. These approaches leverage education, visual feedback, and simulations of critical situations to improve body self-perception.

These studies largely focus on health outcomes to determine the efficacy of VR treatments. While they reported positive clinical effects over a variety of VR experiences, they often pay limited attention to the nature of the hardware and software used. Furthermore, the VR therapies usually relied on custom virtual environments. However, the literature often lacks commentary on the design and development of them. Despite these limitations, VR-based treatments for treating fear-related and anxiety disorders appear to be the most established clinical applications of VR.

Another key area has been in the use of VR for pain management. The mechanism of VR pain control is primarily thought to be distractive, although the precise mode of action remains uncertain [6,42,43]. For example, VR has been used to manage acute pain during in-hospital treatments for burn patients [5,44-47]. Using VR for needle-stick pain has also been researched [6,48]. In the field of chronic pain, VR has also been applied [8,49-51]. Several researchers have explored VR use to treat phantom limb pain [52-55]. VR allows clinicians to present patients with a virtual representation of their missing limb. Through perception and motor training, patients experienced relief from phantom limb pain by seeing their virtual limb move in accordance to their voluntary motor signals.

In the rehabilitation field, there has also been great interest in pairing assistive technologies (eg, robotics, treadmills, wheelchair on rollers, wearable sensors) and VR. The primary goals have been the development of tools that support patient motivation to engage with rehabilitation and to leverage the logistical advantages of digital technology, namely performance monitoring, telehealth, and patient self-management. Additionally, translating physiotherapy exercises and activity training into the VR space allows for much greater control over and variety of scenarios.

For example, robot-assisted upper limb therapy paired with VR visual feedback allows for graded exercises contextualized in a videogame environment [56-58]. In wheelchair simulators, VR enables users to practice wheelchair navigation skills in more dangerous situations such as traffic crossings and crowds without risk [59,60].

Clinical VR research to date has generally been positive, but overall research in this field is in the early stages and faces technical and theoretical hurdles. Most studies have used non-standardized techniques and tools in small-scale pilot studies. Over the last 4 years, the authors have conducted several clinical VR research projects [6,8,49] and found a number of challenges in the field that may limit the validity and generalizability of the work.

## Challenges

### Theoretical Ambiguity and Immaturity

As with the development of any new discipline, establishing a sound theoretical basis and standards is key to the growth of the field. However, there exists some theoretical ambiguity in the field due in part to its immaturity. Overall, VR may be considered as a growing field, defined by both its technology and its effects. The desired effect is to create an immersive experience, whereby the user is placed in a simulated environment that looks and feels as engaging as the real world. The person in this synthetic environment has a specific sense of self-location within it, can move to explore it, feels that the space surrounds them, and can interact with the objects within it. Overall, they feel a sense of presence in this environment, and their actions partially determine what happens within it [61,62].

Technically, the sense of immersion in a VR environment is largely achieved through visual and auditory stimuli that simulate 3D visual and auditory cues available in the real world. Haptic feedback can also contribute to this immersion. Visually, this is delivered to the user via a head-mounted display, which presents the computer-generated imagery (CGI) of the VR scene from the perspective of each of the user’s eyes. The literature suggests that immersion is largely influenced by both visual and audio qualities, although a universally accepted definition is yet to emerge [63-67]. Immersion has been defined as the extent to which a user feels present in the CGI environment, rather than in their actual physical environment [68,69]. In computer science, immersion has more often been defined in terms of the technology and by the extent to which the computer is able to deliver an inclusive, extensive, surrounding, and vivid illusion of reality to the senses of the participant [67]. Therefore, immersion is often referenced by technical considerations, such as field of view and positioning of the virtual body in the CGI. The inclusion of stereoptic imagery is widely thought to be the dominant factor that enhances the immersive experience. Other technical factors, such as greater display resolution or increased field of view are also significant [52,70].

Presence, on the other hand, refers to the sense of being within an environment that is generated through technological means [68,71]. It is viewed as the sense of actually being in a constructed world [68,69,71,72]. Two experiential and technology-dependent dimensions are considered to contribute to a sense of presence. The first dimension is vividness, or the production of a sensory-rich, mediated environment. The second is interactivity, defined as a user’s ability to engage with the environment and modify its form or alter events through interaction with it. An analogy would be that you can become *immersed* through the text in a book, but feel a sense of *presence* in the story only when you feel you are actually there experiencing the events.

This differentiation of immersion from presence (which is seen as more of a subjective element) is fairly well established in computer science, but less so in clinical VR research, where the terms are often used interchangeably. For clinical use, a technical definition of immersion is limited, as it ignores the participant as a co-constructor of the experience. Therefore, concepts of presence and telepresence [68] are likely more useful to clinical applications. An immersive virtual environment can be considered to be a computer-generated environment that elicits the user’s sense of presence or “being there.” It can be seen as an environment that produces an esthetic perception connected to the ideal of total immersion in virtual space involving the willing suspension of disbelief [69,71,73]. In clinical contexts, this sense of presence is likely the key element of interest that differentiates the impact of VR from other distractive and cognitive approaches. Assessment tools that separate these aspects, such as the igroup Presence Questionnaire have been developed [74]. However, clinical VR literature rarely discusses these theoretical aspects nor provides robust theoretical explanations of how VR theory applies to the specific problem under investigation. As VR is essentially a technology-mediated phenomenon, this lack of theoretical distinction, between what actually constitutes a VR experience, at the least, makes meaningful comparisons between clinical studies complex.

Adding more complexity is the issue that the actual nature of the effect of VR on the clinical problem of interest is also often unknown. For example, VR environments are hypothesized to reduce pain by mediating cognitive attentional and distractive mechanisms. The use of VR might act directly and indirectly on pain perception in different ways by altering neurological signaling pathways involving attention, emotion, concentration, memory, touch, and the auditory and visual senses. However, there are competing theoretical explanations of pain and the exact mechanisms of how VR may attenuate it remain unclear [48,75-82]. It has been theorized that VR analgesia stems from the neurobiological interactions of areas of the brain that produce analgesic effect by regulating visual, auditory, and touch sensory experiences [80]. Hoffman et al state that VR works predominantly via distraction. Pain requires attentiveness, and humans have been found to have limited controlled attentional resources [83]. The level and impact of the distraction can depend on the level of the immersion—the more immersive the VR, the more effective in reducing pain [84]. Furthermore, using functional magnetic resonance imaging (fMRI) brain scanning, correlation in pain-related brain activity and subjective pain report was reported, thus, demonstrating the impact of VR on pain-related brain activity in all five regions of the brain [83].

VR has been shown to alter the sense of an individual’s presence to that of being in a virtual world, therefore changing features of the individuals experience associated with sensory affective and cognitive processes.

The validity of clinical VR research also needs to be considered in the context of the theory development process. Overall, there are five major processes that occur in the development and establishment of a theory: (1) creating conceptual meaning, (2) structuring and generalizing the theory, (3) generating the theoretical relationships, (4) applying the theory, and then (5) theory validation by testing in different real-world applications [85]. At this stage of VR development for clinical use, the underpinning theory has yet to reach the higher levels of established validity.

### Standardized Implementation

The type of VR technology implemented varies greatly between clinical studies. It is arguable that the current state of the art is very much technologically led rather than theoretically led, with each new iteration of clinical research using the latest VR applications and hardware with disparate approaches for a variety of clinical conditions. As the hardware and software continue to advance rapidly, studies even a year apart may be using completely different hardware or software and, in many cases, the technology is only vaguely defined [6,12,42].

### Three-Dimensional Versus Two-Dimensional

Many clinical studies have used the term VR to describe significantly different multimedia technologies, including two-dimensional (2D) video screen presentations, 2D-rendered images presented on screens [86] and head-mounted displays [9,46,87], 360-degree 2D presentations on head-mounted displays [88], or computer-assisted virtual environment (CAVE) room-scale projection systems [89,90]. Others used 3D-rendered VR in motion-tracked stereoscopic head-mounted displays, with a wide field of view [8,9,91-93]. There are similar differences in audio use in these studies, with some using positional stereo sound (ie, location-specific sound that moves as the user moves their head) and others using non-spatial audio. Although health outcomes may be comparable, the nature and value of 3D versus 2D applications have not been widely explored in clinical applications.

### Study Design

In addition to the theoretical issues, the nature of VR study design itself represents another significant hurdle. Systematic reviews/meta-analyses illustrate that many of these studies are statistically underpowered, although positive statistical results are frequently claimed [6,12,18,94-96]. To establish clinical efficacy of a therapy, large-scale quality randomized controlled trials are required. Comparative clinical studies also require a suitable control environment to contrast with the VR experience. Few studies make an adequate attempt to address this and frequently neglect to differentiate the effects of the media from the medium itself (both theoretically and in practice). For example, the medium of VR could be the use of VR technology and a head-mounted display to render a 360-degree stereoscopic and stereo audio environment with which a person can interact. The media may be a puzzle-solving interactive VR computer game, a VR rollercoaster ride, or a 3D-rendered high-definition video experience of a beach environment. Failing to explore if it is the VR experience itself or the medium used that is eliciting an effect is problematic. A good design will contrast a VR experience with a non-VR equivalent of the same experience, controlling for the effects of the medium compared to the media. These issues likely reflect some degree of confirmation bias among researchers, but this illustrates the need to implement larger-scale high-quality clinical VR studies.

### Usability and Technical Proficiency

Another more practical challenge faced by clinical researchers is the usability of VR systems and the level of technical proficiency required to run them. Although current VR iterations are designed to be more user friendly, significant technical limitations remain. The use of head-mounted displays is problematic for some patients. They are cumbersome, particularly for patients with head or neck injuries, or for those who are particularly susceptible to eye strain. Additionally, VR applications are generally not usable by people with cognitive or significant visual deficiencies, as they are unable to access existing VR interfaces. Also, prolonged exposure to a screen a few centimeters from the eyes often leads to eye strain or headaches and represents an ongoing issue with VR systems [97,98]. Users with limited head or neck mobility often reported the systems were uncomfortable to use [8]. Furthermore, most advanced head-mounted displays have a cable tether that can be a distraction from the experience or a tripping hazard for older patients.

Cybersickness, as a side effect of VR, is also well documented and limits use by many patients, particularly those taking medications that can cause nausea [8,99-103]. Newer systems that operate at room-scale (ie, where the user can walk around in a pre-determined area) have addressed this to some extent, but many patients also have limited mobility and must use the system in a seated position. This gives rise to another problem: most VR applications are currently designed to be used as either room-scale or seated, with few working well in both configurations. The issue derives from the fact that room-scale VR navigation affords the user much greater range of motion to physically approach virtual items, while the seated position requires a set visual height, longer reaching movements, and controller-based navigation of the environment. The environment design and implementation requirements generally do not transfer well from seated to room-scale and vice versa. Many VR systems have implemented teleportation navigation systems to support moving through larger distances to overcome this issue, but again those designed for room scale use do not adjust well to use from a seated position.

The design and game paradigm of many VR experiences itself can also prove challenging for older patients. For users who have grown up with computer games, the nature of VR experiences is more readily understandable: traversing 3D-rendered worlds, using menus, navigating levels, storing and retrieving items, saving progress, solving game puzzles, and relating button-presses to abstract actions are all mechanics learned through experience. This alignment of VR with recreational gaming is exemplified by the marketing and delivery of HTC Vive and Oculus Rift VR applications through the Steam online gaming platform. Most clinical users are likely to be older adults, who have no such videogame literacy and often find learning these elements frustrating and distracting to their VR experience. Little work exists exploring the VR preferences of these users and the VR market is firmly dominated by the younger consumer.

### Lab Versus In Vivo Practical Issues

Much of the existing VR research has taken place in lab or clinic settings. These environments can be optimized for VR systems. However, much remains to be known about the effects of regular and prolonged VR use for real-world and home applications, where they will be used for many chronic conditions. Certainly, there are common challenges for research requiring any kind of at-home technology implementation such as logistics, remote technical support, learning curve, and compliance. However, there are a few unique challenges to consider in the implementation of VR systems outside the lab.

Current VR systems require dedicated space and are susceptible to interference. Room-scale systems require a 5 ft^2^ space, which may be intrusive to a patient’s living space. Cables may pose tripping hazards. Infrared sensors, such as those used by systems such as the HTC Vive, may be interfered with by devices such as TV remotes, resulting in display cutting out, choppy visuals, and loss of tracking, thus disrupting the user’s experience. Other environmental factors that disrupt infrared tracking, such as climate and reflection of light off windows or mirrors, can be easily mitigated in a lab setting but can be more difficult to cope with in a home. Furthermore, calibration for motion tracking of VR equipment is sensitive and thus movement of equipment must be minimized. Effective installation of VR equipment while still maintaining the usability of the home space is challenging and may be further complicated if there are pets or children in the home.

For clinical research, where a study may take weeks or months, these technological burdens are important to negotiate with participants in advance. Despite these challenges, our experience has shown that research participants are often enthusiastic and willing to accommodate the various needs of the equipment and research study. However, these attitudes may not necessarily carry over to commercial or non-research contexts.

### Costs

Finally, the cost of VR still presents a challenge to implementing large-scale trials [11]. Although costs of head-mounted displays are dropping, quality VR environments still require high-end computer systems with advanced graphics processing to run them. VR applications are also expensive to develop. The current cost of a full system to run a quality VR clinical experience is around US $2,500 per unit plus maintenance costs, making clinical research with multiple users costly. As with any information technology, attrition of value is also rapid; newer technologies rapidly make older systems obsolete. A practical assumption of minimal resale value of a VR system after 3 years is not unreasonable.

## Conclusions

Although clinical VR research looks promising, significant theoretical and practical challenges remain, such as theoretical ambiguity and immaturity, lack of technical standards, differentiating effects of media versus medium, value of 2D versus 3D applications, study design, usability, conducting in vivo research, and economic feasibility. Defining the impact of presence in clinical VR studies and differentiating the concept of presence from immersion (as they are often used synonymously) is a problem, and current research designs are often ill-equipped to differentiate the role of VR from confounding factors. More robust study designs contrasting VR experience with an equivalent non-VR control are required.

Practical challenges also remain, as existing high-end VR systems remain cumbersome and require technical proficiency to use. VR systems are not always user-friendly for patients. Moreover, issues of eye and neck strain and cybersickness remain as practical barriers to wider use. For those undertaking clinical VR research, it is important to keep these issues in mind during efforts to improve the evidence base for these technologies as health interventions.

## Abbreviations

2D: two-dimensional
3D: three-dimensional
CGI: computer-generated imagery
PTSD: posttraumatic stress disorder
VR: virtual reality
